# Biofertilizers function as key player in sustainable agriculture by improving soil fertility, plant tolerance and crop productivity

**DOI:** 10.1186/1475-2859-13-66

**Published:** 2014-05-08

**Authors:** Deepak Bhardwaj, Mohammad Wahid Ansari, Ranjan Kumar Sahoo, Narendra Tuteja

**Affiliations:** 1 Plant Molecular Biology Group, International Centre for Genetic Engineering and Biotechnology (ICGEB), Aruna Asaf Ali Marg, New Delhi 110067, India

**Keywords:** Biofertilizer, Crop improvement, Environmental stress, Mode of action of biofertilizers, Sustainable agriculture

## Abstract

Current soil management strategies are mainly dependent on inorganic chemical-based fertilizers, which caused a serious threat to human health and environment. The exploitation of beneficial microbes as a biofertilizer has become paramount importance in agriculture sector for their potential role in food safety and sustainable crop production. The eco-friendly approaches inspire a wide range of application of plant growth promoting rhizobacteria (PGPRs), endo- and ectomycorrhizal fungi, cyanobacteria and many other useful microscopic organisms led to improved nutrient uptake, plant growth and plant tolerance to abiotic and biotic stress. The present review highlighted biofertilizers mediated crops functional traits such as plant growth and productivity, nutrient profile, plant defense and protection with special emphasis to its function to trigger various growth- and defense-related genes in signaling network of cellular pathways to cause cellular response and thereby crop improvement. The knowledge gained from the literature appraised herein will help us to understand the physiological bases of biofertlizers towards sustainable agriculture in reducing problems associated with the use of chemicals fertilizers.

## Introduction

Conventional agriculture plays a significant role in meeting the food demands of a growing human population, which has also led to an increasing dependence on chemical Fertilizers and pesticides [1]. Chemical fertilizers are industrially manipulated, substances composed of known quantities of nitrogen, phosphorus and potassium, and their exploitation causes air and ground water pollution by eutrophication of water bodies [2]. In this regard, recent efforts have been channelized more towards the production of ‘nutrient rich high quality food’ in sustainable comportment to ensure bio-safety. The innovative view of farm production attracts the growing demand of biological based organic fertilizers exclusive of alternative to agro-chemicals [3]. In agriculture, encourage alternate means of soil fertilization relies on organic inputs to improve nutrient supply and conserve the field management [4]. Organic farming is one of such strategies that not only ensures food safety but also adds to the biodiversity of soil [5]. The additional advantages of biofertilizers include longer shelf life causing no adverse effects to ecosystem [6].

Organic farming is mostly dependent on the natural microflora of the soil which constitutes all kinds of useful bacteria and fungi including the arbuscular mycorrhiza fungi (AMF) called plant growth promoting rhizobacteria (PGPR). Biofertilizers keep the soil environment rich in all kinds of micro- and macro-nutrients via nitrogen fixation, phosphate and potassium solubalisation or mineralization, release of plant growth regulating substances, production of antibiotics and biodegradation of organic matter in the soil [7]. When biofertilizers are applied as seed or soil inoculants, they multiply and participate in nutrient cycling and benefit crop productivity [8]. In general, 60% to 90% of the total applied fertilizer is lost and the remaining 10% to 40% is taken up by plants. In this regard, microbial inoculants have paramount significance in integrated nutrient management systems to sustain agricultural productivity and healthy environment [9]. The PGPR or co-inoculants of PGPR and AMF can advance the nutrient use efficiency of fertilizers. A synergistic interaction of PGPR and AMF was better suited to 70% fertilizer plus AMF and PGPR for P uptake. Similar trend were also reflected in N uptake on a whole-tissue basis which shows that 75%, 80%, or 90% fertilizer plus inoculants were significantly comparable to 100% fertilizer [10]. This review is intended to cater to the needs of agriculturists and plant biologists whose work focuses on creating clean and efficient means to improve the quality of soil by nourishing and maintaining the useful and natural flora of microorganisms or PGPRs. Further, it presents recent developments in the area of field management that reveals the potential application of biofertilizers and increased nutrient profiles, plant growth and productivity, and improved tolerance to environmental stress with a particular emphasis on mechanism of the feat of biofertilizers.

### The microbiome: potential significance of beneficial microbes in sustainable agriculture

The rhizosphere, which is the narrow zone of soil surrounding plant roots, can comprise up to 10^11^ microbial cells per gram of root [11] and above 30,000 prokaryotic species [12] that in general, improve plant productivity [12]. The collective genome of rhizosphere microbial community enveloping plant roots is larger compared to that of plants and is referred as microbiome [13], whose interactions determine crop health in natural agro-ecosystem by providing numerous services to crop plants *viz.,* organic matter decomposition, nutrient acquisition, water absorption, nutrient recycling*,* weed control and bio-control [14]. The metagenomic study provides the individual the core rhizosphere and endophytic microbiomes activity in *Arabidopsis thaliana* using 454 sequencing (Roche) of 16S rRNA gene amplicons [15]. It has been proposed that exploiting tailor-made core microbiome transfer therapy in agriculture can be a potential approach in managing plant diseases for different crops [16]. Rhizosphere microbial communities an alternative for chemical fertilizers has become a subject of great interest in sustainable agriculture and bio-safety programme.

A major focus in the coming decades would be on safe and eco-friendly methods by exploiting the beneficial micro-organisms in sustainable crop production [17]. Such microorganisms, in general, consist of diverse naturally occurring microbes whose inoculation to the soil ecosystem advances soil physicochemical properties, soil microbes biodiversity, soil health, plant growth and development and crop productivity [18]. The agriculturally useful microbial populations cover plant growth promoting rhizobacteria, N_2_-fixing cyanobacteria, mycorrhiza, plant disease suppressive beneficial bacteria, stress tolerance endophytes and bio-degrading microbes [8]. Biofertilizers are a supplementary component to soil and crop management traditions *viz.,* crop rotation, organic adjustments, tillage maintenance, recycling of crop residue, soil fertility renovation and the biocontrol of pathogens and insect pests, which operation can significantly be useful in maintaining the sustainability of various crop productions [19]. *Azotobacter*, *Azospirillum*, *Rhizobium*, cyanobacteria, phosphorus and potassium solubilising microorganisms and mycorrhizae are some of the PGPRs that were found to increase in the soil under no tillage or minimum tillage treatment [20,21]. Efficient strains of *Azotobacter*, *Azospirillum, Phosphobacter* and *Rhizobacter* can provide significant amount of nitrogen to *Helianthus annus* and to increase the plant height, number of leaves, stem diameter percentage of seed filling and seed dry weight [22]. Similarly, in rice, addition of *Azotobacter*, *Azospirillum* and *Rhizobium* promotes the physiology and improves the root morphology [23].

**Figure 1:**
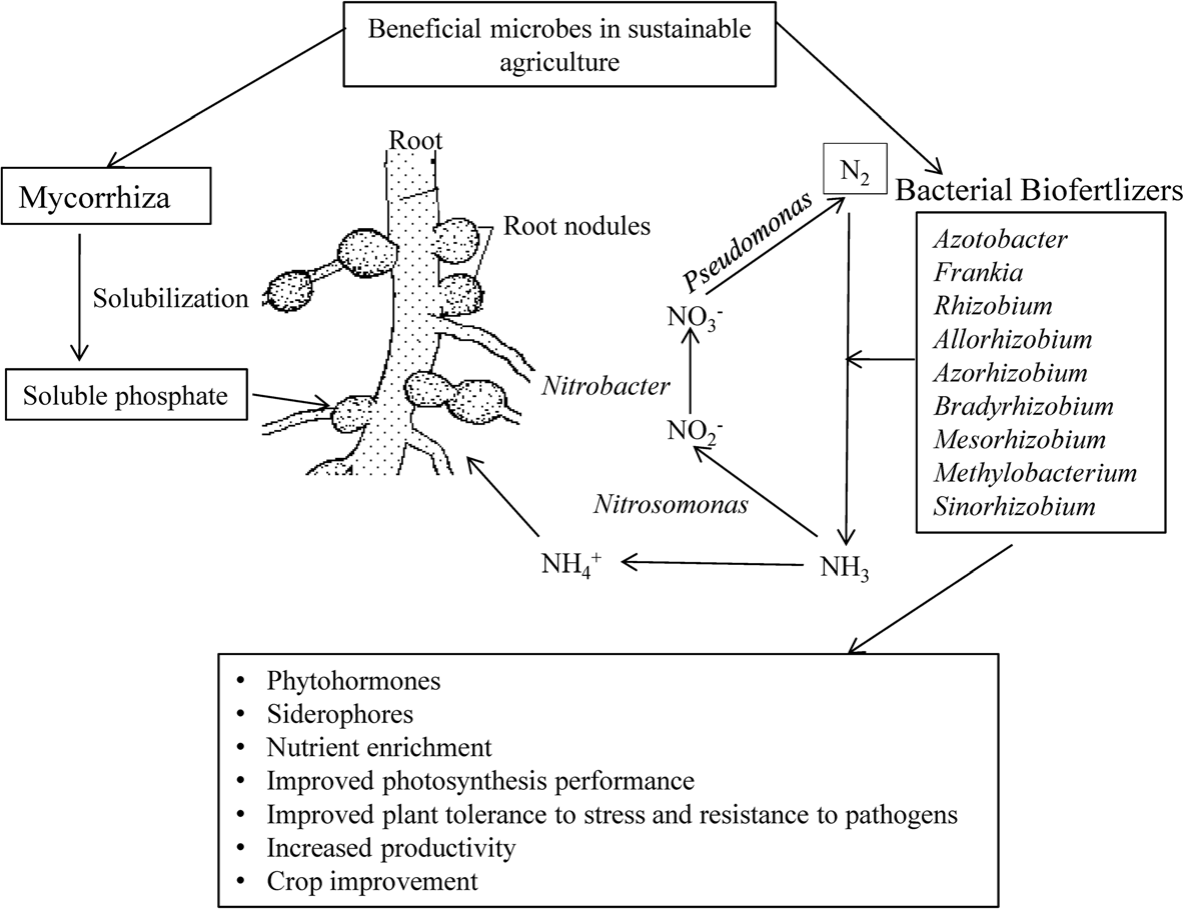
**Potential use of soil microbes in sustainable crop production.** The beneficial soil micro-organisms sustain crop production either as biofertilizers [19] or symbiont [17]. They perform nutrient solubilisation which facilitate nutrient availability and thereby uptake [20,21]. It improves the plant growth by advancing the root architecture [26]. Their activity provides several useful traits to plants such as increased root hairs, nodules and nitrate reductase activity and [36]. Efficient strains of *Azotobacter*, *Azospirillum, Phosphobacter* and *Rhizobacter* can provide significant amount of available nitrogen through nitrogen cycling [22]. The biofertilizers produced plant hormones, which include indole acetic acid (IAA), gibberellins (GA) and cytokinins (CK) [25,44]. Biofertilizers improve photosynthesis performance to confer plant tolerance to stress [44] and increase resistance to pathogens [45] thereby resulting in crop improvement [18].

*Azotobacter* plays an important role in the nitrogen cycle in nature as it possesses a variety of metabolic functions [18]. Besides playing role in nitrogen fixation, *Azotobacter* has the capacity to produce vitamins such as thiamine and riboflavin [24], and plant hormones *viz.,* indole acetic acid (IAA), gibberellins (GA) and cytokinins (CK) [25]. *A. chroococcum* improves the plant growth by enhancing seed germination and advancing the root architecture [26] by inhibiting pathogenic microorganisms around the root systems of crop plants [27]. This genus includes diverse species, namely, *A. chroococcum, A.vinelandii, A. beijerinckii, A. nigricans, A. armeniacus* and *A. paspali*. It is used as a biofertilizer for different crops *viz.,* wheat, oat, barley mustard, seasum, rice, linseeds, sunflower, castor, maize, sorghum, cotton, jute, sugar beets, tobacco, tea, coffee, rubber and coconuts [28]. *Azospirillum* is another free-living, motile, gram variable and aerobic bacterium that can thrive in flooded conditions [6] and promotes various aspects of plant growth and development [29]. *Azospirillum* was shown to exert beneficial effects on plant growth and crop yields both in greenhouse and in field trials [30]. Diverse species of the genus *Azospirillum* including *A. lipoferum*, *A. brasilense*, *A. amazonense*, *A. halopraeferens* and *A. irakense* have been reported to improve productivity of various crops [6]. Interestingly, it was observed that *Azospirillum* inoculation can change the root morphology via producing plant growth regulating substances [31] via siderophore production [6]. It also increases the number of lateral roots and enhances root hairs formation to provide more root surface area to absorb sufficient nutrients [32]. This improves the water status of plant and aids the nutrient profile in the advancement of plant growth and development [33,34]. Co-inoculation of *Azospirillium brasilense* and *Rhizobium meliloti* plus 2,4D posed positive effect on grain yield and N,P,K content of *Triticum aestivum*[35]. *Rhizobium* has been used as an efficient nitrogen fixer for many years. It plays an important role in increasing yield by converting atmospheric nitrogen into usable forms [36]. Being resistant to different temperature ranges *Rhizobium* normally enters the root hairs, multiplies there and forms nodules [37]. *Rhizobium* inoculants in different locations and soil types were reported to significantly increase the grain yields of bengal gram [38], lentil [39], pea, alfalfa and sugar beet rhizosphere [40], berseem [41], ground nut [36] and soybean [42]. These *Rhizobium* isolates obtained from wild rice have been reported to supply nitrogen to the rice plant to promote growth and development [43]. One of the species of *Rhizobium*, *Sinorhizobium meliloti* 1021 infects plants other than leguminous plants like rice to promote growth by enhancing endogenous level of plant hormone and photosynthesis performance to confer plant tolerance to stress [44]. In groundnut, IRC-6 strain of *Rhizobium* has resulted in the enhancement of several useful traits such as increased number of pink coloured nodules, nitrate reductase activity and leghaemoglobin content in 50 DAI (days after inoculation) [36]. Rhizobial symbiosis provides defence to plants against pathogens and herbivores, such as example, Mexican bean beetle [45] and the green house whitefy *Trialeurodes vaporariorum*[46] (Figure 1).

### Biofertlizers exploitation and nutrients profile of crops

A key advantage of beneficial microorganisms is to assimilate phosphorus for their own requirement, which in turn available as its soluble form in sufficient quantities in soil. *Pseudomonas, Bacillus, Micrococcus, Flavobacterium, Fusarium, Sclerotium, Aspergillus* and *Penicillium* have been reported to be active in the solubilisation process [47]. A phosphate-solubilizing bacterial strain NII-0909 of *Micrococcus* sp. has polyvalent properties including phosphate solubilisation and siderophore production [48]. Similarly, two fungi *Aspergillus fumigatus* and *A. Niger* were isolated from decaying cassava peels were found to convert cassava wastes by the semi-solid fermentation technique to phosphate biofertilizers [49]. *Burkholderia vietnamiensis,* stress tolerant bacteria, produces gluconic and 2-ketogluconic acids, which involved in phosphate solubilisation [50]. *Enterobacter* and *Burkholderia* that were isolated from the rhizosphere of sunflower were found to produce siderophores and indolic compounds (ICs) which can solubilize phosphate [51]. Potassium solubilising microorganisms (KSM) such as genus *Aspergillus, Bacillus* and *Clostridium* are found to be efficient in potassium solubilisation in the soil and mobilize in different crops [52]. Mycorrhizal mutualistic symbiosis with plant roots satisfies the plant nutrients demand [53], which leads to enhance plant growth and development, and protect plants from pathogens attack and environmental stress [54]. It leads to the absorption of phosphate by the hyphae from outside to internal cortical mycelia, which finally transfer phosphate to the cortical root cells [55]. Nitrogen fixing cyanobacteria such as *Aulosira, Tolypothrix, Scytonema, Nostoc, Anabaena* and *Plectonema* are commonly used as biofertilizers [56,57]. Besides the contribution of nitrogen, growth-promoting substances and vitamins liberated by these algae *Cylindrospermum musicola* increase the root growth and yield of rice plants [58]. Interestingly, genetic engineering was used to improve the nitrogen fixing potential of *Anabaena* sp. strain PCC7120 [59]. Constitutive expression of the *hetR* gene driven by a light-inducible promoter enhanced HetR protein expression, leading to higher nitrogenase activity in *Anabaena* sp. strain PCC7120 as compared with the wild-type strain. This in turn caused better growth of paddy when applied to the fields [60].

### Biofertilizers relevance and plant tolerance to environmental stress

Abiotic and biotic stresses are the major constraints that are affecting the productivity of the crops. Many tools of modern science have been extensively applied for crop improvement under stress, of which PGPRs role as bio protectants has become paramount importance in this regard [61]. *Rhizobium trifolii* inoculated with *Trifolium alexandrinum* showed higher biomass and increased number of nodulation under salinity stress condition [41,62]. *Pseudomonas aeruginosa* has been shown to withstand biotic and abiotic stresses [63]. Paul and Nair [64] found that *P. fluorescens* MSP-393 produces osmolytes and salt-stress induced proteins that overcome the negative effects of salt. *P. putida* Rs-198 enhanced germination rate and several growth parameters *viz.,* plant height, fresh weight and dry weight of cotton under condition of alkaline and high salt via increasing the rate of uptake of K^+^, Mg^2+^ and Ca^2+^, and by decreasing the absorption of Na^+^[65]. Few strains of *Pseudomonas* conferred plant tolerance via 2,4-diacetylphloroglucinol (DAPG) [66]. Interestingly, systemic response was found to be induced against *P. syringae* in *Arabidopsis thaliana* by *P. fluorescens* DAPG [67]. Calcisol produced by PGPRs *viz., P. alcaligenes* PsA15, *Bacillus polymyxa* BcP26 and *Mycobacterium phlei* MbP18 provides tolerance to high temperatures and salinity stress [68]. It has been demonstrated that inoculation of plant with AM fungi also improves plant growth under salt stress [69]. *Achromobacter piechaudii* was also shown to increase the biomass of tomato and pepper plants under 172 mM NaCl and water stress [70]. Interestingly, a root endophytic fungus *Piriformospora indica* was found to defend host plant against salt stress [69]. In one of the studies it was found that inoculation of PGPR alone or along with AM like *Glomus intraradices* or *G. mosseae* resulted in the better nutrient uptake and improvement in normal physiological processes in *Lactuca sativa* under stress conditions. The same plant treated with *P. mendocina* increased shoot biomass under salt stress [71]. Mechanisms involved in osmotic stress tolerance employing transcriptomic and microscopic strategies revealed a considerable change in the transcriptome of *Stenotrophomonas rhizophila* DSM14405^T^ in response to salt stress [72]*.* Combination of AM fungi and N_2_-fixing bacteria helped the legume plants in overcoming drought stress [73]. Effect of *A.brasilense* along with AM can be seen in other crops such as tomato, maize and cassava [74-76]. *A. brasilense* and AM combination improved plant tolerance to various abiotic stresses [77]. The additive effect of *Pseudomonas putida* or *Bacillus megaterium* and AM fungi was effective in alleviating drought stress [78]. Application of *Pseudomonades sp.* under water stress improved the antioxidant and photosynthetic pigments in basil plants. Interestingly, combination of three bacterial species caused the highest CAT, GPX and APX activity and chlorophyll content in leaves under water stress [79]. *Pseudomonas* spp. was found to cause positive affect on the seedling growth and seed germination of *A. officinalis* L. under water stress [80]. Photosynthetic efficiency and the antioxidative response of rice plants subjected to drought stress were found to increase after inoculation of arbuscular mycorrhiza [81]. The beneficial effects of mycorrhizae have also been reported under both the drought and saline conditions [82]. Heavy metals such as cadmium, lead, mercury from hospital and factory waste accumulate in the soil and enter plants through roots [83]. *Azospirillium* spp, *Phosphobacteria* spp and *Glucanacetobacter* spp. isolated from rhizosphere of rice field and mangroves were found to be more tolerant to heavy metal specially iron [83,84]. *P. potida* strain 11 (P.p.11), *P. potida* strain 4 (P.p.4) and *P. fluorescens* strain 169 (P.f.169) can protect canola and barley plants from the inhibitory effects of cadmium via IAA, siderophore and 1-aminocyclopropane-1-carboxylate deaminase (ACCD) [85]. It was reported that rhizoremediation of petroleum contaminated soil can be expedited by adding microbes in the form of effective microbial agent (EMA) to the different plant species such as cotton, ryegrass, tall fescue, and alfalfa [86].

PGPRs as biological agents proved to be one of the alternatives of chemical agents to provide resistance to against various pathogen attacks [87]. Apart from acting as growth-promoting agents they can provide resistance against pathogens by producing metabolites [88]. *Bacillus subtilis* GBO3 can induce defense-related pathways *viz.,* salicylic acid (SA) and jasmonic acid (JA) [89]. Application of PGPR isolates *viz., B. amyloliquefaciens* 937b and *B. pumilus* SE-34 provide immunity against tomato mottle virus [90]. *B. megaterium* IISRBP 17, characterized from stem of black pepper, acts against *Phytophthor capsici*[91]. *Bacillus subtilis* N11 along with mature composts was found to control *Fusarium* infestation on banana roots [92]. Similarly, *B. subtilis* (UFLA285) was found to provide resistance against *R. solani* and also it induced foliar and root growth in cotton plants [93]. In another interesting study *Paenibacillus polymyxa* SQR-21 was identified as a potential agent for the bio-control of *Fusarium* wilt in watermelon [94]. Further, the exploitation of PGPRs was found to be effective to manage the spotted wilt viruses in tomato [87], cucumber mosaic virus of tomato and pepper [90], and banana bunchy top virus in banana [95]. In some cases it was shown that along with bacteria, mycorrhizae can also confer resistant against fungal pathogens and inhibit the growth of many root pathogens such as *R. solani*, *Pythium* spp., *F. Oxysporum, A. obscura and H. annosum*[96,97] by improving plant nutrients profile and thereby productivity [69]. For instance *Glomus mosseae* was effective against *Fusarium oxysporum* f. sp. Basilica which causes root-rot disease of basil plants [98]. *Medicago tranculata* also showed induction of various defense-related genes with mycorrhizal colonization [99]. It was shown that addition of arbuscular mycorrhizal fungi and *Pseudomonas fluorescens* to the soil can reduce the development of root-rot disease and enhance the yield of *Phaseolus vulgaris* L. [100].

### Mechanism of action of various biofertilizers

Mycorrhiza is the association of fungus with the roots of higher plants. While it remains an enigma, it serves as a model system to understand the mechanism behind stimulation of growth in the root cells as a result of mycorrhizal inhabitation. Genome sequencing of two EM fungi (ectomycorrhizae), the *L. bicolor* 13, and *T. melanosporum* (black truffle) 14, helps in the identification of factors that regulate the development of mycorrhiza and its function in the plant cell [101]. Fifteen genes that up-regulated during symbiosis were identified as putative hexose transporters in L. *bicolor.* Its genome lacked genes encoding invertases making it dependent on plants for glucose. However, *melanosporum* possesses one invertase gene, and unlike *L. bicolor* it can directly use the sucrose of the host [101]. The up-regulation of transporter genes during symbiosis indicated the action of transportation of useful compounds like amino acids, oligopeptides and polyamines through the symbiotic interface from one organism to other. Free living mycelium can take nitrate and ammonium from the soil. Subsequently, these compounds reach the mantle and hartig net and are then transferred to the plants. Cysteine-rich proteins (MISSP7) of fungus play an important role as effectors and facilitators in the formation of symbiotic interfaces [102]. Many genes related to auxin biosynthesis and root morphogenesis showed up-regulation during mycorrhizal colonization [69,103,104]. Further, *G. versiforme* possesses inorganic phosphate (Pi) transporters on its hyphae which help in the direct absorption of phosphate from the soil and a glutamine synthase gene was found in *G. intraradice*, which strengthens the possibility of nitrogen metabolism in fungal hyphe that can be transported later to the plant [105]. Bioactive compounds called Myc factors similar to Nod factors of *Rhizobium* are suggested to be secreted by mycorrhiza and *Rhizobium* and perceived by host roots for the activation of signal transduction pathway or common symbiosis (SYM) pathway [106,107]. The pathways that prepare plant for both AM and *Rhizobium* infection have some common points. The common SYM pathway prepares the host plant to bring about changes at the molecular and anatomical level with the first contact of fungal hyphae. So far, calcium is supposed to be the hub of secondary messengers via Ca^2+^ spiking in the nuclear region of root hairs [108]. *Rhizobium leguminosarum* biovar *viciae* can induce various genes in the plants like pea, alfalfa and sugar beet as evident from the microarray studies [40]. PGPR produce IAA which, in turn, induces the production of nitric Oxide (NO), which acts as a second messenger to trigger a complex signaling network leading to improved root growth and developmental processes [109].

Expression of ENOD11 and many defense-related genes and root remodelling genes get up-regulated during entry. Subsequently, this allows the formation of a pre-penetration apparatus or PPA [110]. Though the biology behind the development of arbuscules is unknown, a gene called vapyrin when knocked down causes a decline in the growth of arbuscules [111]. Many other genes including subtilisin protease 65, phosphate transporter 66 or two ABC transporters 67 are known to be involved in arbuscules formation [112,113]. Nitrogen-fixation genes are popularly used by scientists today to create engineered plants that can fix atmospheric nitrogen. The induction of *nif* genes in case of nitrogen fixing bacteria takes place under low concentration of nitrogen and oxygen in the rhizosphere [1]. Interestingly, sugarcane plantlets inoculated with a wild strain of *G. diazotrophicus*, have demonstrated fixation of radioactive N_2_ when compared with the *G. diazotrophicus* mutant that has mutant *nif* D gene which proved the significance of *nif* genes. Efficiency of nitrogen fixation is dependent on the utilization of carbon [114,115]. A bacterium like *Bacillus subtilis* (UFLA285) can differentially induce 247 genes in cotton plant as compared to control where no PGPR was supplied to the cotton plant [85]. Many disease resistance genes that work via jasmonate/ethylene signaling as well as osmotic regulation via proline synthesis genes were differentially expressed with UFLA285 induction [85]. Various differentially expressed genes were identified which include metallothionein-like protein type 1, a NOD26-like membrane integral protein, ZmNIP2-1, a thionin family protein, an oryzain gamma chain precursor, stress-associated protein 1 (OsISAP1), probenazole-inducible protein PBZ1 and auxin and ethylene-responsive genes [116]. The expression of the defense-related proteins PBZ1 and thionins were found to get repressed in the rice–H seropedicae association, suggesting the modulation of plant defense responses during colonisation [116].

**Figure 2:**
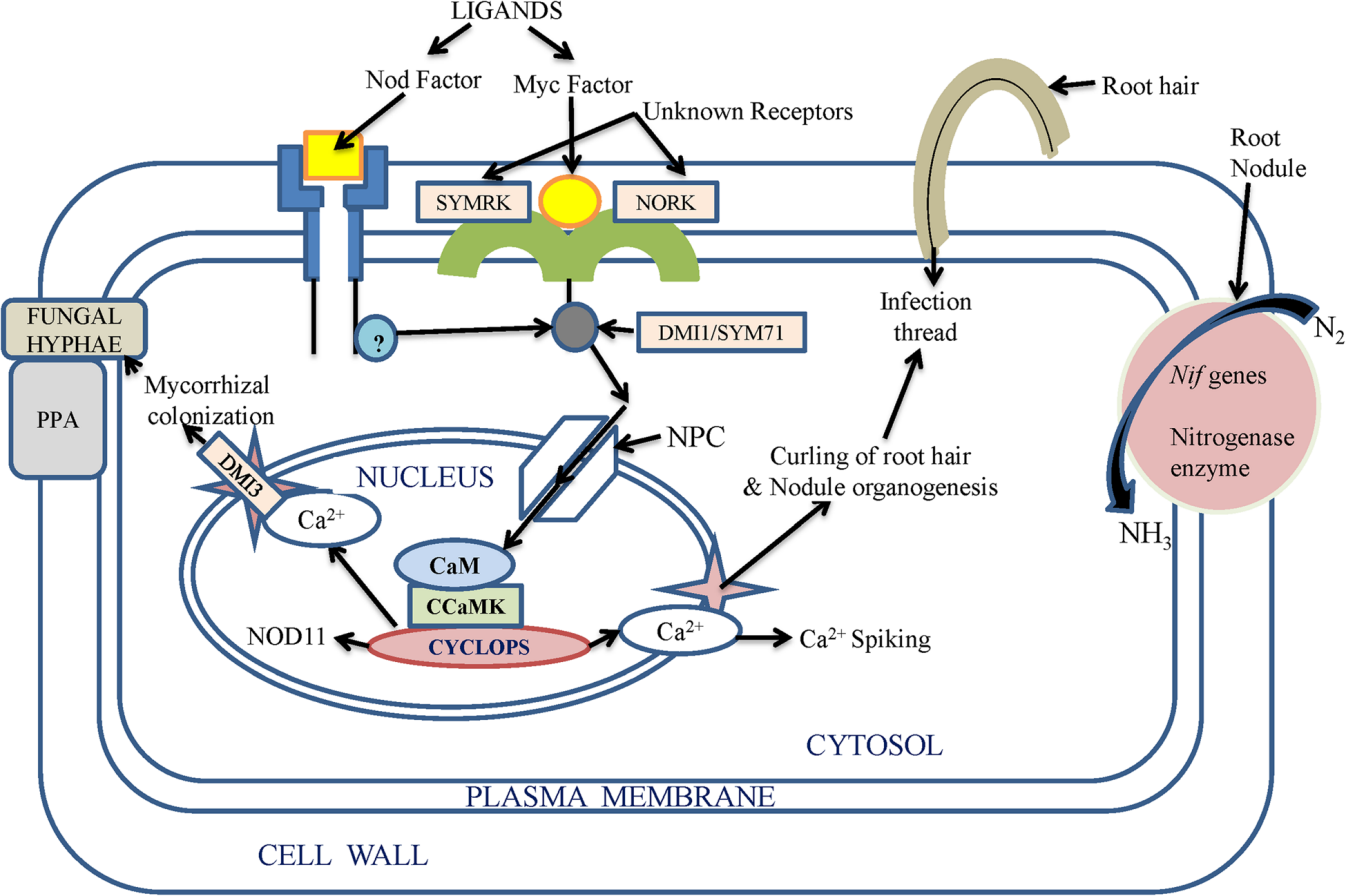
**Hypothetical mechanism of action of biofertilizers in the root cell.** Bioactive ligands called Myc factors and Nod factors secreted by mycorrhiza and *Rhizobium* were perceived by host roots to trigger the signal transduction pathway [106,107], which initiates further signal transduction pathway through unknown receptors (SYMRK and NORK) [101] which trigger release of Ca^2+^ in the cytosol [108]. The whole pathway involves receptor like kinases or other kinase related proteins like DMI and SYM71 to phosphorylate their substrates [123,124]. Nuclear pore complex (NPC) and some of its proteins (NUP) play role in calcium spiking. DM1 proteins play role in maintaining periodic oscillation of calcium ions inside and outside the nucleus. Several channels proteins (Ca^2+^channel proteins) also facilitate this process with the help of various transporters [108]. *CCaMK* is a calcium calmodulin-dependent protein kinase, which phosphorylate the product of CYCLOPS protein thus initiating activation of various genes involving formation of structures like nodule and (PPA) pre-penetration apparatus [124].

Among the PGPR species, *Azospirillum* was suggested to secrete gibberellins, ethylene and auxins [117]. Some plant associated bacteria can also induce phytohormone synthesis, for example lodgepole pine when inoculated with *Paenibacillus polymyxa* had elevated levels of IAA in the roots [118]. *Rhizobium* and *Bacillus* were found to synthesize IAA at different cultural conditions such as pH, temperature and in the presence of agro waste as substrate [119]. Ethylene, unlike other phytohormones, is responsible for the inhibition of growth of dicot plants [69]. It was found by Glick *et al*. [120] that PGPR could enhance the growth of plant by suppressing the expression of ethylene. Interestingly, a model was suggested in which it was shown that ethylene synthesis from 1-aminocyclopropane-1-carboxylate (ACC), an immediate precursor of ethylene, which is hydrolyzed by bacterial ACC-deaminase enzyme in the need of nitrogen and carbon source is also one of the mechanisms of induction of conditions suitable for growth. ACC-deaminase activity was also found in the bacteria such as *Alcaligenes sp., Bacillus pumilus, Pseudomonas sp*. and *Variovorax paradoxus*[69]. The involvement of ACC deaminase in the indirect influence on the growth of plants was proved in Canola, where mutations in ACC deaminase gene caused the loss of effect of growth promoting *Pseudomonas putida*[29]. Interestingly, the potential of PGPRs was further enhanced by introducing genes involved in the direct oxidation (DO) pathway and mineral phosphate solubilisation (MPS) into some useful strains of PGPRs. Gene encoding glucose dehydrogenase (gcd) involved in the DO pathway was cloned and characterized from *Acinetobacter calcoaceticus* and *E. coli* and *Enterobacter asburiae*[121]. Also a soluble form of gcd has been cloned from *Acinetobacter calcoaceticus* and *G. oxydans*[122]. Furthermore there are reports of site-directed mutagenesis of glucose dehydrogenase (GDH) and gluconate dehydrogenase (GADH) that has improved the activity of this enzyme. Mere substitution of S771M provided thermal stability to *E.coli* while mutation of glutamate 742 to lysine improved the EDTA tolerance of *E. coli* PQQGDH. The application of this technology was achieved by transferring genes involved in the DO pathway *viz., GDH, GADH* and pyrroloquinoline quinine (*PQQ*) to rhizobacteria, and phosphoenolpyruvate carboxylase (*PPC*) to *P. Fluorescens*, provide the MPS trait [122] (Figure 2).

## Conclusions

Environmental stresses are becoming a major problem and productivity is declining at an unprecedented rate. Our dependence on chemical fertilisers and pesticides has encouraged the thriving of industries that are producing life-threatening chemicals and which are not only hazardous for human consumption but can also disturb the ecological balance. Biofertilizers can help solve the problem of feeding an increasing global population at a time when agriculture is facing various environmental stresses. It is important to realise the useful aspects of biofertilizers and implement its application to modern agricultural practices. The new technology developed using the powerful tool of molecular biotechnology can enhance the biological pathways of production of phytohormones. If identified and transferred to the useful PGPRs, these technologies can help provide relief from environmental stresses. However, the lack of awareness regarding improved protocols of biofertiliser applications to the field is one of the few reasons why many useful PGPRs are still beyond the knowledge of ecologists and agriculturists. Nevertheless, the recent progresses in technologies related to microbial science, plant-pathogen interactions and genomics will help to optimize the required protocols. The success of the science related to biofertilizers depends on inventions of innovative strategies related to the functions of PGPRs and their proper application to the field of agriculture. The major challenge in this area of research lies in the fact that along with the identification of various strains of PGPRs and its properties it is essential to dissect the actual mechanism of functioning of PGPRs for their efficacy toward exploitation in sustainable agriculture.

## Competing interests

Authors declare that they have no competing interests.

## Authors’ contributions

MWA and DB contributed with the paper writing, data researching and designs the Figures. RKS supported the paper writing, data researching and revised the changes made to this paper. NT approved the changes made, and also with data researching and formatted the review. All authors read and approved the final manuscript.
